# Handgrip Strength has Declined Among Adults, Particularly Males, from Shanghai Since 2000

**DOI:** 10.1186/s40798-024-00800-x

**Published:** 2024-12-23

**Authors:** Dao Wang, Yang Liu, Justin J. Lang, Marilyn G. Klug, Ryan McGrath, Grant R. Tomkinson

**Affiliations:** 1 Physical Fitness Research and Health Guidance Center, Shanghai Research Institute of Sports Science (Shanghai Anti-Doping Agency), Shanghai, China; 2 School of Physical Education, Shanghai University of Sport, Shanghai, China; 3 Shanghai Research Center for Physical Fitness and Health of Children and Adolescents, Shanghai, China; 4 Faculty of Medicine, School of Epidemiology and Public Health, University of Ottawa, Ottawa, ON Canada; 5 Alliance for Research in Exercise, Nutrition and Activity (ARENA), Allied Health and Human Performance, University of South Australia, GPO Box 2471, Adelaide, SA 5001 Australia; 6 Department of Population Health, University of North Dakota, Grand Forks, ND USA; 7 Healthy Aging North Dakota (HAND), North Dakota State University, Fargo, ND USA; 8 Department of Health, Nutrition, and Exercise Sciences, North Dakota State University, Fargo, ND USA; 9 Fargo VA Healthcare System, Fargo, ND USA; 10 Department of Geriatrics, University of North Dakota, Grand Forks, ND USA

**Keywords:** Muscle strength, Adult, Public health, Physical fitness, Health status

## Abstract

**Background:**

Handgrip strength (HGS) is an excellent marker of general strength capacity and health among adults. We aimed to calculate temporal trends in HGS for adults from Shanghai between 2000 and 2020.

**Methods:**

Adults aged 20–59 years from Shanghai, China, were included. Representative cross-sectional HGS data (*n* = 127,756) were collected in 2000, 2005, 2010, 2014, and 2020. HGS was measured using isometric dynamometry and was adjusted for body size (i.e., height-squared). Trends in mean adjusted HGS were calculated using general linear models with adjustments for age, sex, location, occupation, blood pressure, and exercise time. Trends in distributional characteristics were described visually and calculated as the ratio of coefficients of variation (CVs).

**Results:**

We found a significant, small decline in mean adjusted HGS (effect size (ES) [95%CI]: −0.21 [−0.22, −0.20]) since the year 2000. Negligible temporal differences were found across age, location, and occupation groups, with a 2.8-fold greater decline for men than for women. Overall, distributional variability declined negligibly (ratio of CVs [95% CI]: 0.92 [0.91, 0.93]). We also observed a negligible trend (ES < 0.20) in distributional asymmetry among adults with low adjusted HGS (below the 25th percentile) and a small decline (ES = 0.20−0.49) in adults with high adjusted HGS (above the 75th percentile).

**Conclusions:**

There was a recent small decline in adjusted HGS for adults from Shanghai, which was greater for men than for women and nonuniform across the population. A decline in adjusted HGS may represent a decline in the general/functional health of the population.

**Supplementary Information:**

The online version contains supplementary material available at 10.1186/s40798-024-00800-x.

## Key Points

- Mean body size-adjusted handgrip strength (HGS) among adults from Shanghai (aged 20–59-years) declined between 2000 and 2020.
- The decline in adjusted HGS was 2.8-fold larger for men than for women, which suggests that the sex gap in adjusted HGS is closing.
- There was a smaller temporal decline for adults with low adjusted HGS than for adults with high adjusted HGS.
- A decline in adjusted HGS may represent a similar decline in the general and functional health of the population.

**Supplementary Information:**

The online version contains supplementary material available at 10.1186/s40798-024-00800-x.

## Background

Muscle strength reflects the capacity of a muscle or group of muscles to produce maximal force [1]. Although muscle strength cannot be defined by a single measure, it is widely characterised by handgrip strength (HGS) using isometric dynamometry in clinical and epidemiological settings [2]. This is because HGS is feasible, reliable and safe to assess [3, 4], and relates very well to other measures of muscle strength [5, 6]. HGS is also significantly linked with present and future health conditions [7–9]. A recent overview of eight systematic reviews on nearly 2 million adults revealed that low HGS was significantly linked with early death from all causes and cardiovascular disease, as well as a higher incidence of physical disability [9]. This health-related evidence supports the recent promotion of muscle strengthening activities using major muscle groups at least twice a week (in addition to aerobic activities) in global [10] and national [11] physical activity guidelines for adults.

National trends in HGS likely reflect trends in general and functional health and may provide insight into the effectiveness of healthy public policies. In a recent meta-analysis of trends in HGS for more than 2.5 million adults from 14 countries between 1960 and 2017, it was found that most countries experienced negligible-to-small declines ranging from a standardized (Cohen’s) effect size (ES) [12] of 0.05 to 0.27 (or 0.6% to 6.3%) per decade after the year 2000 [13]. China was among those countries analysed, and experienced a steady decline of 0.21 ES or 4.0% per decade for 719,885 Chinese adults aged 20–69-years between 2000 and 2014 [13, 14]. Unfortunately, this meta-analysis [13] calculated only broad regional- and national-level trends in mean HGS, and did not calculate trends in either mean HGS adjusted for potential confounders (e.g., body size) or distributional characteristics (i.e., variability and asymmetry). Adjusting HGS for body size results in a more sensitive measure of strength capacity within a population since population subgroups are known to vary in body size (e.g., sex and ethnicity). Knowledge of trends in body size-adjusted HGS (herein called *adjusted HGS*) may shed light on possible mechanistic causes. Furthermore, information on trends in distributional characteristics may help identify whether the trend in HGS was uniform (i.e., symmetric) or nonuniform (i.e., asymmetric) across the population. Understanding temporal trends in adult HGS levels may provide insight for healthy public policies and interventions, identify population subgroups that have tracked poorly over time, and potentially predict future disease burden.

Approximately every 5 years as part of China’s national physical fitness surveillance system [14], the Physical Fitness Research and Health Guidance Center has assessed the HGS of representative samples of 20- to 59-year-old adults from Shanghai, allowing for the analysis of temporal trends. Using these data, we aimed to calculate temporal trends in the means and distributional characteristics of adjusted HGS for adults from Shanghai between 2000 and 2020.

## Methods

### Participants and Sampling Procedures

Using a repeated cross-sectional design and stratified random cluster sampling, representative samples of civilian, noninstitutionalized adults (aged 20–59 years) from the municipality of Shanghai, China, were assessed for strength capacity using HGS in 2000, 2005, 2010, 2014, and 2020. Located in the east of China, Shanghai is one of four direct-administered municipalities (province-level cities) in China. Shanghai has very high human development [15], with more than 25 million people, and is China’s largest (and the world’s third largest) megacity [16].

The sampling procedure for Shanghai is described in the Supplementary Data, Table S1 in Appendix 1, and generally followed that for China’s national physical fitness surveys, which has been described in detail elsewhere [14]. Briefly, the sampling procedure for Shanghai comprised (1) all districts from urban and rural areas, (2) the random selection of streets from districts and towns from counties, (3) the random selection of residential communities, villages, or institutions from selected streets and towns, and (4) the systematic sampling (to ensure equal numbers) of eligible participants from selected residential communities, villages, and institutions who were permanent residents of Shanghai. Each cross-sectional survey was conducted between April and October, and the overall response rate in each cross section was consistently very high (> 90%). Written informed consent was provided by participants, and the National Physical Fitness Surveillance Center of China approved the testing protocols. [17–21]

Prior to sample selection, potential participants self-reported whether they had chronic health conditions that could affect their ability to participate in exercise testing. Overall, HGS was measured for 127,756 apparently healthy adults (49.7% [*n* = 63,535] men, 50.3% [*n* = 64,221] women) aged 20–59 years between 2000 and 2020. Of these, 57.6% (*n* = 73,554) lived in urban areas, and 42.4% (*n* = 54,202) lived in suburban and rural areas. Among urban adults, 49.4% (*n* = 36,317) and 50.6% (*n* = 37,237) were manual and nonmanual laborers, respectively.

### Measures

#### Handgrip Strength and Body Height

HGS was assessed by the Jianmin digital hand dynamometer with the WCS-II model (Beijing Xindong Huateng Sports Facilities Company Ltd., Beijing, China) used in the 2000, 2005, 2010, and 2014 surveys and the GMCS-WCS 3 model (Beijing Xindong Huateng Sports Facilities Company Ltd., Beijing, China) used in the 2020 survey. The dynamometer was adjusted for hand size by ensuring that the middle phalange of each participant’s index finger was flexed to 90° and rested flat atop the handle. A submaximal effort practice trial was performed to ensure that the dynamometer was properly adjusted for hand size and to confirm understanding of the HGS protocol. Participants maximally squeezed the dynamometer with their dominant hand while standing straight, their feet hip width apart, their arms straight and down by their side, and their radioulnar and wrist joints in neutral. Two trials, with no fixed time in between, were performed while being verbally encouraged. HGS was calculated as the better of two trials (in kilograms [kg]).

Standing height (in metres [m]) was measured using a fixed stadiometer with an adjustable headboard (Beijing Xindong Huateng Sports Facilities Company Ltd., Beijing, China). To best remove the influence of body size, we followed the recommendation of Nevill et al. [22] and normalised HGS by standing height-squared (i.e., HGS/height^2^ in kg/m^2^), which is the most appropriate body size dimension associated with HGS identified by allometry. All survey staff underwent a 1-week training course on the use of the standardised protocols and equipment for data collection.

#### Covariates

Participants self-reported their age at last birthday, sex (male or female), and total weekly exercise time. The location (rural or urban) was defined by the sampling area. Occupation (manual labour [e.g., construction worker, factory employee, waiter] or nonmanual labour [e.g., teacher, doctor, office worker]) was classified for urban adults only by survey staff according to the sampling unit (e.g., if the sampling unit was a construction company, then all builders were classified as manual labourers, and all administration staff were classified as nonmanual labourers). Mean arterial pressure (in millimetres of mercury [mmHg])—the average arterial pressure during a single cardiac cycle and a general measure of cardiovascular health [23]—was calculated from objectively measured resting systolic and diastolic blood pressure [24].

### Statistical Analyses

Trends in the mean adjusted HGS were calculated using SAS EG 7.1 (Cary, North Carolina, USA). General linear models (proc GLM) were used to assess trends, with the testing year as the independent variable and adjusted HGS as the dependent variable. Linear models were used because they naturally summarized the overall trends. The models were adjusted for sex, age, location, occupation, mean arterial pressure, and weekly exercise time. We calculated trends for all 20- to 59-year-olds and stratified the trends by sex, age, location, and occupation. Trends were expressed as absolute rates of change (i.e., the regression coefficient), percent rates of change (i.e., the regression coefficient expressed as a percentage of the sample weighted mean), and standardized Cohen’s ES [12] (i.e., the regression coefficient divided by the pooled standard deviation). The magnitudes of the trends in the means were interpreted as negligible (ES < 0.20), small (ES = 0.20–0.49), moderate (ES = 0.50–0.79), and large (ES > 0.80) [12]. Positive trends in means indicated improvements and negative trends in means indicated declines.

Temporal trends in distributional characteristics were examined visually and as trends in the coefficient of variation (CV, the ratio of the standard deviation to the mean). Trends in CVs were analysed as the ratio of CVs by dividing the 2020 CVs by the 2000 CVs using a procedure described elsewhere [25]. Ratios > 1.1 indicated substantial increases in variability (i.e., the magnitude of variability increased in relation to the mean), ratios < 0.9 indicated substantial declines in variability (i.e., the magnitude of variability decreased in relation to the mean), and ratios between 0.9 and 1.1 inclusive indicated negligible trends in variability (i.e., the magnitude of variability did not change substantially in relation to the mean) [26]. Trends in distributional asymmetry were visualised by LOWESS (LOcally WEighted Scatterplot Smoother) curves (tension = 66) [27], by plotting the overall trends in adjusted HGS from the 1st to the 99th percentiles.

## Results

With adjustment for covariates, we found a significant small decline in adjusted HGS (trend in means [95% CI]: −0.70 kg/m^2^ [−0.74, −0.65], −5.3% [−5.6, −4.9], or −0.21 ES [−0.22, −0.20]) between 2000 and 2020 (Table 1). We found a small sex-related temporal difference, with a 2.8-fold greater decline for men (trend in means [95% CI]: −1.08 kg/m^2^ [−1.15, −1.02], −6.9% [−7.3, −6.5], −0.44 ES [−0.47, −0.41]) than for women (trend in means [95% CI]: −0.31 kg/m^2^ [−0.36, −0.26], −2.9% [−3.4, −2.4], −0.16 ES [−0.19, −0.13]). Age-, location-, and occupation-related temporal differences were negligible.

**Table 1:** Temporal trends in means and variability for body size-adjusted handgrip strength among 20- to 59-year-old adults from Shanghai between 2000 and 2020

Group	*n*	Mean ± SD (kg/m^2^)	Trends in means (95%CI)			Trends in variability (95%CI)
			Absolute (kg/m^2^)	Percent (%)	Standardized (ES)	Ratio of CVs
*All*						
20–59 years	127,756	13.2 ± 3.3	–0.70 (–0.74, –0.65)	–5.3 (–5.6, –4.9)	–0.21 (–0.22, –0.20)	0.92 (0.91, 0.93)
*Sex*						
Men	63,535	15.7 ± 2.5	–1.08 (–1.15, –1.02)	–6.9 (–7.3, –6.5)	–0.44 (–0.47, –0.41)	0.94 (0.93, 0.96)
Women	64,221	10.8 ± 1.9	–0.31 (–0.36, –0.26)	–2.9 (–3.4, –2.4)	–0.16 (–0.19, –0.13)	0.90 (0.88, 0.91)
*Age*						
20–39 years	63,417	13.2 ± 3.3	–0.97 (–1.03, –0.91)	–7.3 (–7.8, –6.9)	–0.29 (–0.31, –0.27)	0.94 (0.92, 0.96)
40–59 years	64,339	13.2 ± 3.2	–0.45 (–0.51, –0.39)	–3.4 (–3.8, –2.9)	–0.14 (–0.16, –0.12)	0.90 (0.88, 0.91)
*Location*						
Rural	54,202	13.4 ± 3.3	–1.08 (–1.15, –1.01)	–8.1 (–8.6, –7.5)	–0.33 (–0.35, –0.31)	0.94 (0.92, 0.96)
Urban	73,554	13.1 ± 3.3	–0.46 (–0.52, –0.41)	–3.5 (–3.9, –3.1)	–0.14 (–0.16, –0.13)	0.91 (0.89, 0.92)
*Occupation*						
Manual	36,317	13.0 ± 3.2	–0.55 (–0.63, –0.48)	–4.3 (–4.8, –3.7)	–0.17 (–0.19, –0.15)	0.91 (0.89, 0.93)
Nonmanual	37,237	13.2 ± 3.3	–0.37 (–0.45, –0.29)	–2.8 (–3.4, –2.2)	–0.11 (–0.14, –0.09)	0.90 (0.88, 0.92)

Positive trends in means indicated improvements in means and negative trends in means indicated declines; the magnitudes of the trends in means were interpreted as negligible (ES < 0.20), small (ES = 0.20–0.49), moderate (ES = 0.50–0.79), and large (ES > 0.80); a ratio of CVs > 1.1 indicated substantial increases in variability, ratios < 0.9 indicated substantial declines in variability, and ratios between 0.9 and 1.1 indicated negligible trends in variability. Trends stratified by occupation were only available for adults from urban areas. The mean ± SD body size-adjusted handgrip strength values (kg/m^2^) are also shown

*kg/m*^*2*^ Kilograms per metre-squared, *%* percent, *SD* Standard deviation, *CV* Coefficient of variation, *n* Sample size, *95% CI* 95% Confidence interval, *ES* Effect size

**Fig. 1:**
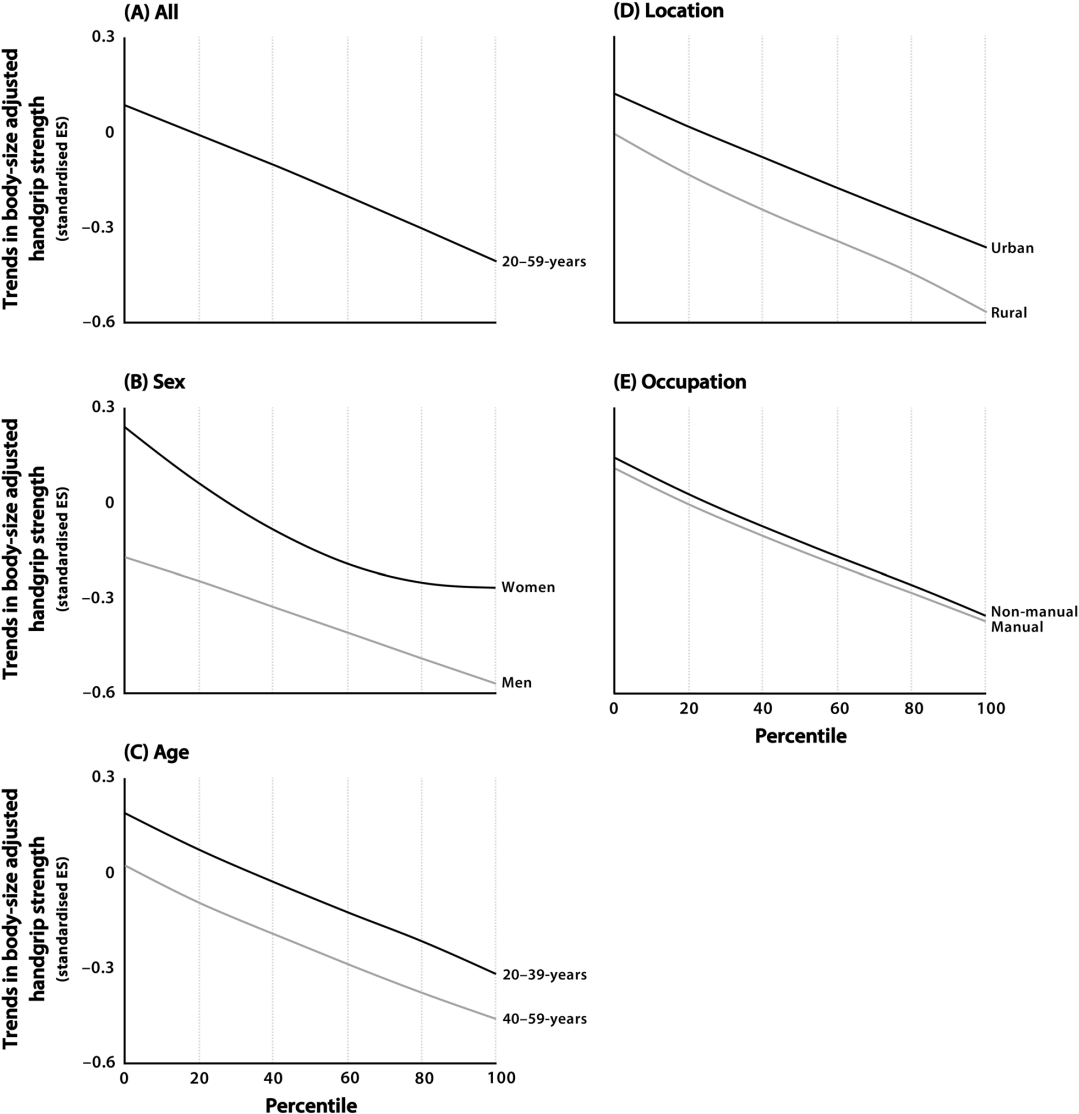
Distributional trends in body size-adjusted handgrip strength for 20- to 59-year-old adults from Shanghai between 2000 and 2020. Notes: Distributional trends are shown for (**A**) all 20- to 59-year-olds and for different sex (**B**), age (**C**), location (**D**), and occupation (**E**) groups; trends between 2000 and 2020 are shown as standardized ES, with positive trends indicating improvements and negative trends indicating declines; the solid lines are the LOWESS (LOcally WEighted Scatterplot Smoother) curves (tension = 66), which are used to represent the trends at various percentiles (range: 1st to 99th); sloped lines indicate asymmetric trends (e.g., lines that sloped downwards from the top left to the bottom right indicated relatively smaller trends [declines or improvements] in adults with low adjusted handgrip strength than for adults with high adjusted handgrip strength) and flat (horizontal) lines indicate symmetric trends (i.e., uniform trends across all percentiles or adjusted handgrip strength levels). Abbreviations: ES = effect size

We found evidence for nonuniform trends in adjusted HGS. Overall, we found a negligible decline in distributional variability (ratio of CVs [95% CI]: 0.92 [0.91, 0.93]) (Table 1). We also found a negligible trend in distributional asymmetry among those with low adjusted HGS (below the 25th percentile) and a small decline in those with high adjusted HGS (above the 75th percentile) (Fig. 1). The trends in distributional asymmetry differed by sex, but were similar for all age, location, and occupation groups. For men, we found a small decline in those with low adjusted HGS and a small-to-moderate decline in those with high adjusted HGS. For women, we found a negligible-to-small improvement in those with low adjusted HGS and a small decline in those with high adjusted HGS.

## Discussion

We calculated temporal trends in adjusted HGS for a representative sample of 127,756 adults from Shanghai between 2000 and 2020. Overall, we found a small decline in mean adjusted HGS, a small sex-related temporal difference, a negligible decline in distributional variability, and a smaller decline in those with low vs. high adjusted HGS, which reflected a trend toward distributional asymmetry. The closing of the sex gap in adjusted HGS may reflect the recent promotion of gender equality policies and programs, and the better trends for those with low adjusted HGS are important because this population subgroup is at greater risk of early all-cause death and physical disability than other subgroups. Additional health- and fitness-enhancing efforts are required to further reduce these declines and to improve population-level strength capacity.

Few studies have formally examined temporal trends in adult HGS. The most comprehensive synthesis is a recent meta-analysis of trends in mean HGS for 2.5 million adults from 14 countries between 1960 and 2017, which revealed that most countries had experienced negligible-to-small (per decade) declines after the year 2000 [13]. National trends for Chinese adults aged 20–59 years indicate a small (per decade) decline in mean HGS from 2000 to 2014 [13, 14], which is twice as large as the decline we observed in our findings. This temporal difference may reflect differences in participant pools (national vs. megacity), the span of measurement years (2000–14 vs. 2000–20), the operationalisation of HGS (absolute HGS vs. body size-adjusted HGS), and statistical adjustments for other potential confounders. Several studies have found corresponding trends in mean HGS and body size (operationalized as standing height and body mass [28] or body mass index (BMI) [29]), and others have found opposing trends [30, 31]. Two recent studies have, however, reported trends in mean adjusted HGS. Feng et al. [32] reported a decline in mean body mass-adjusted HGS for a nationally-representative sample of 712,442 Chinese adults aged 20–69-years between 2000 and 2014. Similarly, Dodds et al. [30] reported a decline in mean BMI-adjusted HGS for a broadly representative sample of English adults aged 50–89-years between 2004 and 2012 [30]. A decline in adjusted HGS may be the result of long-term exposure to high body size, which is associated with low HGS later in life possibly due to the chronic effects of inflammation or insulin resistance [33]. Trends in body composition may also be involved [34]. Like Dodds et al. [30] who additionally adjusted for trends in self-reported physical activity levels, our findings suggest that trends in adult HGS are likely influenced by factors other than trends in body size and self-reported exercise time. Because muscle-strengthening activity (e.g., resistance activity) positively influences strength capacity [35], it is possible that the observed decline in mean HGS among Shanghai adults reflected a decline in muscle-strengthening activity participation. Strategies that promote participation in muscle-strengthening activities (e.g., the 2021 physical activity and sedentary behaviour guidelines for Chinese people, which recommend muscle-strengthening activities [in addition to aerobic activities] on two or more days a week for adults) [36], might be a suitable population approach to improving strength capacity levels among adults.

Our finding of a smaller decline in mean adjusted HGS among women than men suggests that the sex gap in strength capacity is closing among adults from Shanghai. In contrast, most studies have found negligible temporal differences in strength capacity between men and women [13, 25, 30, 37, 38], with one study on 3890 older Swiss adults finding a widening of the sex gap as seen by no change in HGS for men and a decline for women between 2005 and 2015 [29]. It is not clear whether this sex-related difference among adults from Shanghai is reflected nationally because studies using nationally representative data on Chinese adults have not formally compared trends among men and women [14, 32]. Our complementary trends in distributional asymmetry analysis indicated that the relatively smaller decline in mean adjusted HGS for Shanghai women was driven by the negligible-to-small improvement in women with low adjusted HGS, which would have boosted mean adjusted HGS values in recent years. Perhaps the recent promotion of gender equality policies and programs that aim to empower women [39–42] has helped close the sex gap in adjusted HGS through increased leisure-time physical activity and physical activity in other domains. There is some evidence to support this, as national physical fitness surveillance data indicate that the increase in the percentage of 20- to 59-year-old Chinese adults achieving recommended leisure-time physical activity levels between 2000 and 2014 was significantly greater for women than for men [14]. Although sex equity has not been fully achieved, China, like most other countries, seems to be performing better in this respect today than in previous decades [43].

Our formal analysis of trends in distributional variability, coupled with our visual analysis of trends in distributional asymmetry, indicated that the decline in mean adjusted HGS was not uniform across the population, with trends better for those with low adjusted HGS than for those with high adjusted HGS. This finding suggested that certain factors have differentially affected population subgroups. Studies reporting trends in the distributional characteristics of adult HGS are rare. A recent study, which examined trends in the distributional variability of adult HGS over a similar period and with a similar statistical approach as in our study, revealed a substantial decline in the variability of HGS for a nationally representative sample of 176,449 older Japanese adults aged 65–79 years between 1998 and 2017 [25]. Unlike in the present study where we found a trend in distributional asymmetry for adjusted HGS across the population, it was uncertain whether older Japanese adults experienced a similar trend in distributional asymmetry. Future studies should examine the underlying mechanistic factors and population health consequences of these distributional trends in strength capacity among adults from Shanghai.

Using standardized testing protocols and a consistent sampling strategy, we calculated temporal trends in adjusted HGS for a representative sample of adults from Shanghai over a 20-year period. Our trends in mean values were adjusted for potential confounders, and we additionally calculated trends in distributional characteristics that have rarely been reported in the literature. Despite these strengths, our study had limitations. Although the overall response rate in each survey year was similar and very high, our trends may have been biased if there were trends in the percentage of adults who either opted out or were medically excluded. Unfortunately, no such trend data were available. Despite our best efforts to adjust our trends for potential confounders, it is possible that our trends were biased by residual confounding.

## Conclusions

Since the turn of the century there has been a small decline in adjusted HGS for 20- to 59-year-old adults from Shanghai. We found a small sex-related temporal difference, with a greater decline for men than for women, which suggests that the sex gap in strength capacity is closing. We also found distributional trends in adjusted HGS, as evidenced by a smaller decline in adults with low adjusted HGS than in adults with high adjusted HGS. While declining levels of strength may translate to declining levels of general and functional health, our finding of smaller declines among adults with low adjusted HGS is encouraging for public health in Shanghai because these individuals have the highest risk of early all-cause mortality and physical disability. We recommend fitness-enhancing (especially muscle-strengthening) policies and programs to further reduce these declines and to improve population strength capacity levels. Future studies should examine temporal trends in the means and distributional characteristics of other physical fitness measures among adults from Shanghai, other administrative divisions, and nationally to confirm our findings. Shanghai’s continued surveillance of adult fitness levels, which inform China’s national physical fitness surveillance system, provides important insight into population-level trends in functional health and fitness and we hope encourages other areas of the world to engage in a similar cost-effective public health surveillance strategy.

## Supplementary Information

Additional file 1.

## Data Availability

The datasets used and/or analysed during the current study are available from the first author upon reasonable request.

## Abbreviations

95% CI: Ninety-five percent confidence interval
Adjusted HGS: Body size-adjusted handgrip strength
CV: Coefficient of variation
ES: Standardized (Cohen’s) effect size
GLM: General linear model
HGS: Handgrip strength (HGS)
kg: Kilogram
m: Metre
mmHg: Millimetres of mercury
