# Supplementary material for: Handgrip Strength has Declined Among Adults, Particularly Males, from Shanghai Since 2000
Source: Sports Med Open. 2024 Dec 23;10:132. doi: 10.1186/s40798-024-00800-x (PMC11666846; doi:10.1186/s40798-024-00800-x)
Supplement: Supplementary file 1 — Additional file 1. [file 40798_2024_800_MOESM1_ESM.docx]

## APPENDIX 1

**Table S1.** Sampling procedure for the fitness surveillance of 20- to 59-year-old adults from Shanghai between 2000 and 2020.

|  | **2000** | **2005** | **2010** | **2014** | **2020** |
| --- | --- | --- | --- | --- | --- |
| Sampling districts | All districts of Shanghai | All districts of Shanghai | All districts of Shanghai | All districts of Shanghai | All districts of Shanghai |
| Age (years) | 20–59 | 20–59 | 20–59 | 20–59 | 20–59 |
| Domicile | Permanent residence in Shanghai | Permanent residence in Shanghai | Permanent residence in Shanghai | Permanent residence in Shanghai | Permanent residence in Shanghai |
| Sample requirement | Apparently healthy, no obvious physiological conditions | Apparently healthy, no obvious physiological conditions | Apparently healthy, no obvious physiological conditions | Apparently healthy, no congenital or hereditary diseases, no acute or chronic diseases | Apparently healthy, no congenital or hereditary diseases, no acute or chronic diseases |
| Sample category | Urban (manual labour, nonmanual labour) and rural | Urban (manual labour, nonmanual labour) and rural | Urban (manual labour, nonmanual labour) and rural | Urban (manual labour, nonmanual labour) and rural | Urban (manual labour, nonmanual labour) and rural |
| Sample group and size | 5-year age groups (20–24, 25–29, 30–34, 35–39, 40–44, 45–49, 50–54, 55–59) with a sample size of *n* = 40 per sex-age group | 5-year age groups (20–24, 25–29, 30–34, 35–39, 40–44, 45–49, 50–54, 55–59) with a sample size of *n* = 40 or 80 (depending on the district) per sex-age group | 5-year age groups (20–24, 25–29, 30–34, 35–39, 40–44, 45–49, 50–54, 55–59) with a sample size of *n* = 50 or 100 (in Minhang District) per sex-age group | 5-year age groups (20–24, 25–29, 30–34, 35–39, 40–44, 45–49, 50–54, 55–59) with a sample size of *n* = 50 or 100 (in Minhang District) per sex-age group | 5-year age groups (20–24, 25–29, 30–34, 35–39, 40–44, 45–49, 50–54, 55–59) with a sample size of *n* = 50 or 100 (in Minhang District) per sex-age group |
| Sampling method | Stratified random cluster sampling | Stratified random cluster sampling | Stratified random cluster sampling | Stratified random cluster sampling | Stratified random cluster sampling |
| Registered resident population in Shanghai | 13.216 million | 13.603 million | 14.123 million | 14.387 million | 14.756 million |
| Permanent population in Shanghai | 16.086 million | 18.903 million | 23.027 million | 24.671 million | 24.884 million |
